# CAG Student Prize Paper – A4 EXPLORING THE EFFECTS OF COMMONLY USED ARTIFICIAL FOOD COLOURANTS IN THE PATHOGENESIS OF COLITIS

**DOI:** 10.1093/jcag/gwaf042.004

**Published:** 2026-02-13

**Authors:** T Seto, J Grondin, H Wang, A Jamal, W Khan

**Affiliations:** McMaster University, Hamilton, ON, Canada; McMaster University, Hamilton, ON, Canada; McMaster University, Hamilton, ON, Canada; McMaster University, Hamilton, ON, Canada; McMaster University, Hamilton, ON, Canada

## Abstract

**Background:**

Inflammatory bowel diseases (IBDs) are serious chronic inflammatory conditions of the gastrointestinal (GI) tract. While the pathogenesis of IBD is not fully understood, it is well established that genetic susceptibility, immune dysregulation, the gut microbiota composition, and environmental factors collectively drive disease progression. Although significant research progress has been made in identifying IBD-susceptibility genes and elucidating the roles of the immune system and gut microbiota in IBD pathogenesis, limited advancements have been made in defining specific environmental factors that impact these conditions. Emerging evidence suggests diet, particularly the overconsumption of processed foods, is linked with IBD. One factor by which the diet may affect colitis is the presence of food additives in the food supply. Among various food additives that are used, artificial food colourants (AFC) play a key role in enhancing the appearance of food and attracting consumers. Recently, we have shown that chronic exposure to the commonly used AFC, Allura Red (Red 40), increases susceptibility to colitis in experimental models of colitis. However, the effects of other AFCs, such as Tartrazine (TZ), Sunset Yellow (SY), and Brilliant Blue (BB), on the GI tract have not been extensively explored.

**Aims:**

To investigate if chronic exposure to the common AFCs, TZ, SY, and BB increases the host’s susceptibility to colitis.

**Methods:**

C57BL/6 SPF mice were treated with TZ, SY, BB, or no dye in their drinking water for a period of 12 weeks. Colitis was induced using 3.5% dextran sulfate sodium (DSS) for 7 days. During DSS treatment, mice were continuously exposed to their respective dye. Disease activity index (DAI), which incorporates body weight, stool consistency, and fecal bleeding was assessed during DSS administration. Intestinal inflammation was further assessed by checking macroscopic scores, histological damage scores, and various pro-inflammatory cytokines in the colonic tissues by ELISA.

**Results:**

BB-treated mice revealed significant increases in DAI, macroscopic score, histological score, colonic IL-1β, IL- 6, TNF-α, IL-17, and fecal lipocalin-2 compared to non-dye treated control mice. SY-treated mice showed increased DAI, macroscopic, and histological score compared to controls, however colonic IL-1β, IL- 6, TNF-α, IL-17, and fecal lipocalin-2 were non-significant. All evaluations on inflammatory parameters involving TZ-treated mice yielded non-significant results.

**Conclusions:**

These findings suggest that chronic exposure to BB increases susceptibility to colitis. Defining the effects of AFCs on intestinal inflammation will not only advance our understanding of how environmental factors contribute to IBD pathogenesis, but it may also add to the growing body of knowledge prompting scrutiny of the use of AFCs in the food industry

**Funding Agencies:**

CIHR

